# A Novel Interaction between Tryptophan Hydroxylase 2 (TPH2) Gene Polymorphism (rs4570625) and BDNF Val^66^Met Predicts a High-Risk Emotional Phenotype in Healthy Subjects

**DOI:** 10.1371/journal.pone.0162585

**Published:** 2016-10-03

**Authors:** Maeson S. Latsko, T. Lee Gilman, Lindsey M. Matt, K. Maria Nylocks, Karin G. Coifman, Aaron M. Jasnow

**Affiliations:** Department of Psychological Sciences, Kent State University, Kent, OH, 44242, United States of America; Chiba Daigaku, JAPAN

## Abstract

Poor inhibitory processing of negative emotional content is central to many psychiatric disorders, including depression and anxiety. Moreover, increasing evidence suggests that core aspects of emotion-inhibitory processing are largely inherited and as such may represent a key intermediate or risk-related phenotype for common affective diseases (e.g., unipolar depressive, anxiety disorders). The current study employed a candidate-gene approach in order to most effectively examine this complex behavioral phenotype. We examined the novel interaction between BDNF (Val^66^Met) and TPH2 (rs4570625) polymorphisms and their influence on behavioral inhibition of negative emotion in two independent investigations of healthy adults. BDNF Met carriers consistently report greater symptoms of affective disease and display corresponding behavioral rigidity, while TPH2 T carriers display poor inhibitory processing. These genotypes are traditionally perceived as ‘risk’ genotypes when compared to their respective major Val and G homozygous genotypes, but evidence is mixed. Recent studies in humans and mutant mouse models suggest biological epistasis between BDNF and genes involved in serotonin regulation. Moreover, polymorphisms in the TPH2 gene may have greater influence on serotonergic function than other more commonly studied polymorphisms (e.g., 5-HTTLPR). We observed consistent evidence across two different emotion-inhibition paradigms, one with high internal validity (Study 1, n = 119) and one with high ecological validity (Study 2, n = 115) that the combination of Val/Val and G/G genotypes was clearly associated with impaired inhibition of negative emotional content. This was followed by individuals carrying the BDNF—Met allele (including Met/Val and Met/Met) when combined with the TPH2—T allele (including T/G and T/T combinations). The consistency of these results across tasks and studies suggests that these two groups may be particularly vulnerable to the most common psychiatric disorders and should be targets for future clinical investigation.

## Introduction

Difficulty inhibiting negative emotional content has been consistently linked to specific and highly-impairing emotion-related disorders such as depression [1,2]. Further, impairments in inhibiting negative emotions are broadly associated with symptoms of common affective diseases (such as unipolar depressive, anxiety, and stress disorders) [3], including: patterns of negative self-referential thought [4–6]; enduring negative mood; inattention; impulsivity; agitation and arousal, as well as disruptions in sleep and eating [7–10]. Accordingly, poor inhibition of negative emotional content is now considered a key trans-diagnostic risk factor for affective disease. Here, we sought to evaluate a *novel* interaction between the BDNF Val^66^Met polymorphism and a less commonly studied serotonergic system polymorphism in the tryptophan hydroxylase 2 gene (*TPH2*) on negative emotion inhibition in two independent samples of healthy adults. Persistent risk-related differences in cognitive and emotional processes over a wide range of contexts may be influenced by genetic polymorphism interactions, such as those between BDNF and serotonergic systems [11–13]. Indeed, despite recent reduced enthusiasm for candidate-gene research, there is a clear need for detailed examination of *complex behavioral phenotypes*, work that is by necessity limited to candidate gene studies given the related methodological demands. Genome-wide association studies (GWAS) focused on affective disorders such as major depressive disorder are often limited to distal measurements given the immense sample sizes (tens of thousands) required [14,15]. In fact, despite considerable evidence implicating Val^66^Met in candidate gene research [16,17], multiple GWAS studies have thus far not implicated any BDNF or TPH2 polymorphisms in association with affective disorders [16–21]. Accordingly, here we focused on careful assessment of a novel yet potentially informative interaction between two polymorphisms in these systems in the prediction of a key trans-diagnostic risk factor: poor inhibition of negative emotional content.

TPH2 is the neuron-specific enzyme isoform catalyzing the rate-limiting step in serotonin synthesis [22–25]. Thus, polymorphisms in the TPH2 gene may have the potential for greater influence on serotonergic function than other identified and highly studied polymorphisms within this system (e.g., 5-HTTLPR). One common SNP within *TPH2* is a T substitution for G in the 5′regulatory region of the gene (rs4570625; also noted as G-703T). The T allele of rs4570625 is hypothesized to be associated with reduced serotonin levels [26,27], and has been linked to deficits in executive attention and may be more common in human samples with ADHD and obsessive-compulsive disorder; two disorders characterized by poor inhibitory processes [28–30]. *TPH2* variation has been linked to common affective disorders such as major depression in a recent meta-analysis [31] and is predictive of amygdala responses to emotional content [28,32–34]. Indeed, evidence points to the relevance of this polymorphism in affective processing as well as higher-order cognitive processes associated with inhibition [35]. Though some research has examined interactions between the *TPH2* rs4570625 polymorphism and the 5-HTTLPR polymorphism [29,36–39], none have examined an interaction between *TPH2* rs4570625 and *BDNF* Val^66^Met on cognitive function and never in relation to emotional content.

The broad influence of BDNF on neural plasticity and development has consistently suggested its key role in emotion-related learning and response patterns. The most widely studied BDNF polymorphism, the Val^66^Met SNP (rs6265) leads to a non-synonymous methionine (Met) substitution for valine (Val) at codon 66 in the prodomain of BDNF. This substitution results in reduced BDNF release under stimulated conditions [40]. The BDNF Val^66^Met Met allele has been associated with impaired fear extinction [41,42] and increased sensitivity to environment [43]. Further, the BDNF Val^66^Met polymorphism influences serotonergic system functioning in humans [44,45]. Animal studies further support reciprocal modulation occurring between serotonergic and BDNF systems [46–50].

Given these previous data suggesting individual contributions of the BDNF Val^66^Met Met allele or TPH2 G-703T T-allele to emotion regulation, we hypothesized these polymorphisms’ interactive influence would be compounded to result in more significant impairment of emotion-inhibitory processing. In particular, recent studies in humans and mutant mouse models suggest biological epistasis between BDNF and another gene involved in serotonin regulation (serotonin transporter, *SLC6A4*) [11,12,46,47,51] that may alter neural circuitry and thereby influence risk for psychiatric disease. For instance, reductions in brain BDNF levels through genetic knockout exacerbate serotonin level deficiencies already observed in serotonin transporter (5-HTT) knockout mice. Reduced BDNF also increases avoidance behavior and neuroendocrine stress responses in these double mutant mice [46,47,51]. Further, TPH2 knockout mice, but not 5-HTT knockouts, exhibit increases in hippocampal BDNF [50], together suggesting a reciprocal feedback system by which BDNF promotes serotonergic activity, while serotonin reduces BDNF activity. These effects are supported by earlier literature demonstrating BDNF-serotonergic interactions *in vitro* [52–56]. Most notable is work by Weinberger and colleagues, [11] in which the Met allele of the BDNF Val^66^Met polymorphism mitigated the 5-HTTLPR S allele-induced reductions in prefrontal cortex gray matter volume and amygdala connectivity. Although counterintuitive, these data suggest that levels of BDNF may protect against the potentially adverse neurodevelopmental effects of increased serotonergic signaling. Indeed, much of the literature recognizes that optimal serotonin function is essential for proper neurodevelopment, and sub- or supra-optimal functioning can shift cortical and limbic circuitry development resulting in lifelong emotional consequences [57–62].

### Current study

In this investigation, we used a candidate gene approach in combination with behavioral measures to examine the interaction between BDNF Val^66^Met and TPH2 G-703T polymorphisms on inhibition of negative emotion. Although candidate gene approaches have been recently criticized because of non-replication issues and an inability to account for polygenic influences on phenotypes, this approach is appropriately suited for the highly detailed behavioral data collected in the present study. Moreover, by testing the predicted relationships across two distinct samples and lab paradigms, we begin to address issues related to reliability and replication.

We focused exclusively on testing the association between patterns of emotion-inhibitory processing and allelic combinations in *healthy* adults. Poor emotion-inhibitory processing is well established in clinical samples with common affective disorders. Individual differences in emotion-inhibition can predict responsivity to treatment or even identify individuals whose symptoms are particularly intractable [63–65]. However, linking specific allelic combinations to similar risk-related patterns in healthy adults might be a first step to understanding how genetic variation contributes to the onset of disease. Indeed, most models of emotion-related psychiatric disease involve complex interactions between inherited and learned/environmental factors. Characteristics influencing vulnerability to common affective disorders vary in heritability, from 20–30% for emotion regulation [66,67], 56% for temperament [68], and up to 99% for executive function [69]. Accordingly, though genes substantially contribute to multiple core aspects of emotion-inhibitory processing, identification of clear and unique gene-disorder relationships is unlikely [70]. In contrast, linking genetic variation to underlying and *more proximal* intermediate phenotypes shows considerable promise [71].

We conducted two studies involving distinct samples of adult participants to determine the combined contributions of these polymorphisms to variability in emotion-inhibitory processing and psychiatric risk. In Study 1 we examined genetic influences on performance during an Emotion-word Stroop task, a well-validated emotion-inhibition paradigm. In Study 2 we evaluated genetic influences on spontaneous and more naturalistic inhibition of negative emotion by indexing real-time down-regulation of negative emotion during emotionally-evocative films [72,73]. Naturalistic emotion-response paradigms are recognized for their ability to capture salient patterns of emotion that more closely reflect real-world responding [74] and are more highly predictive of salient clinical phenomena [65,75,76].

## Methods and Materials

### Ethics statement

The study was approved by the Kent State institutional review board governing human subjects research and has been performed in accordance with the ethical standards laid down in the 1964 Declaration of Helsinki (World Medical Association, 2013). The study also complies with the APA ethical standards for treatment of human subjects. All participants provided written informed consent before participating in the study.

### Subjects

Study 1 and Study 2 were comprised of two independent samples. **Study 1:** Participants were 119 undergraduate students (62% female; 77% Caucasian, 11% African American, 3% Asian; 95% Non-Hispanic). Mean age was *M* = 20.14, *SD* = 4.15. Mean rated depression on the Center of Epidemiological Studies Depression Scale (CES-D) [77] was typical for community samples *M* = 11.74, *SD* = 8.37 (above 16 denotes clinically significant depression; n = 31 scored above 16; range was 0–50). **Study 2:** Participants were 115 undergraduate students (64% female; 79% Caucasian, 12% African American, 3% Asian, 6% Other; 91% Non-Hispanic). Mean age was *M* = 20.80, *SD* = 6.48. Mean rated depression on the CES-D was *M* = 11.54, *SD* = 7.43 (scores above 16 reflect clinically significant depression symptoms; n = 23 scored above 16; range was 1–39).

### Genotyping and RFLP

2 mL of saliva was collected from each participant and was stored at -20°C [78]. DNA extraction was accomplished using prepIT-L2P and following the manufacturer’s recommendations (DNA Genotek inc., Ottawa, Canada). DNA was then purified using Genomic DNA Clean & Concentrator kit (Zymo Research, Irvine, CA) and quantified using SYBR Green I dye (Lonza, Walkersville, MD). Genomic DNA was diluted to 5 ng/μL prior to polymerase chain reaction (PCR). In a final volume of 20 μl, 1 μl DNA was amplified, and each reaction consisted of 0.5 μM forward primer, 0.5 μM reverse primer, 0.5 μl Taq polymerase solution, 5 mM MgCl_2_, 2 mM dNTPs, and 10% glycerol. Both genes were run on an Eppendorf PCR Mastercycler pro (model no. 6321, Hamburg,Germany) using a touchdown PCR cycle protocol adopted from Anchordoquy et al., [79] and modified to have a 65°C annealing temperature for 10 cycles, followed by a 55°C annealing temperature for 35 cycles.

### rs4570625 (G-703T)

Primers used to amplify rs4570625 within the TPH2 gene were adopted from Mössner et al., [80] (Forward: 5'-TTT TAT GAA AGC CAT TAC ACA T; Reverse: 5'-TTC CAC TCT TCC AGT TAT TTT A). Following amplification, a restriction digest was performed to discern the presence or absence of the T to G substitution. 10 μl of the PCR product was used in the total reaction with 1X Cutsmart buffer and 4 U/reaction PsiI enzyme (New England BioLabs, Ipswich, MA) in a total volume of 20 μl and was incubated for 3 hours at 37°C. Restriction fragments were visualized using a 2% agarose gel. In the presence of the T substitution, the product yields 149 and 55 bp lengths. In the absence of the substitution (i.e., G allele), the product was undigested, and a 204 bp fragment was visualized. Ten percent of sample genotypes were separately reconfirmed with 100% concordance.

### BDNF Val^66^Met

Primers used to amplify Val^66^Met polymorphism in the BDNF gene were adopted from Hünnerkopf et al. [81] (Forward: 5’-AAA GAA GCA AAC ATC CGA GGA CAA G; Reverse: 5’- ATT CCT CCA GCA GAA AGA GAA GAG G). Following amplification, a digest similar to above was performed, using NlaIII enzyme (New England BioLabs, Ipswich, MA) incubated at 37°C for 1 hour. Restriction fragments were visualized on a 3% agarose gel. In the presence of methionine substitution (A allele) yielded product lengths of 140, 77, and 57 bp. In the absence of the substitution, (G allele) the PCR product was undigested and a 274 bp product was visualized. Ten percent of sample genotypes were separately reconfirmed with 100% concordance.

### Procedures

In each study, after providing written informed consent, participants provided demographic information, reported current depressive symptoms using the CES-D [82], and provided saliva samples (Oragene-DISCOVER OGR-500 kits). They then proceeded to complete the emotion-inhibitory processing tasks described below.

### Emotion-inhibitory processing assessment.

#### Study 1, Emotion-word Stroop

Participants completed an Emotion-word Stroop [83] task via computer (*ePrime*, *2*.*0;*Psychology Software Tools, Inc., Pittsburgh, PA). The task consisted of 180 trials of negative, positive, or neutral words (60 of each type) and participants were instructed to label the text color (red, green, yellow, or blue) by button press as quickly as possible and to ignore word meaning. Emotion-word Stroops are established indicators of emotion-inhibitory processing as individuals must inhibit the emotion-word content in order to attend to the text color [84,85]. Participants were seated comfortably in front of a computer monitor by themselves and were instructed to “work as quickly as possible while avoiding mistakes”. Participants completed practice trials and then began the task. Words were presented as one set, in random order, and randomized by valence. Before each trial, participants saw a white fixation point on a black screen for 500 ms, followed by the stimulus (affective or neutral word). Stimuli remained on the screen until the participant indicated color of the word. As soon as the participant responded to the stimuli, the fixation point re-appeared on the screen.

Data were cleaned following standard conventions [86]. Overall error rates were low and there were no differences in errors by word type (neutral % error M = 4.38, SD = 0.05; negative % error M = 4.31, SD = 0.05; positive % error M = 4.40, SD = 0.05). One individual was dropped from the analysis because of reaction times greater than 2 SDs from the mean (final *N* = 119). Only reaction times from correct responses were used in the analyses.

#### Study 2, Naturalistic emotion-inhibition

Participants were asked to “emotionally engage” with four previously validated highly emotionally evocative films [72,73], each approximately five minutes in duration, with a brief break between. As is customary when assessing emotion during naturalistic tasks, assessments were repetitive and multi-dimensional (emotion responses were based on both self-report and objective coding of facial emotion) so as to increase validity [65,72]. Films were presented in a sequence to maximize the intensity of elicited responses and to place greater demands on participants to *naturally* inhibit their negative emotion responses from a negative film to a positive film. Intense negative emotions were elicited in the first film clip (anger and disgust: *Road to Guantanamo*, Revolution Films, 2006) and third film clip (sadness: *The Champ*, Metro-Goldwyn Mayer, 1979). Each negative film was followed by an explicitly positive film, as such the second film clip (happiness: *Alive*, Paramount Pictures, 1993) and fourth film clip (amusement: *Between two Ferns*, www.comedyordie.com, 2010) depicted highly positive scenes. Response to the two positive films were aggregated. Given that the film sequence consistently shifted from negative to explicitly positive, inhibition or down-regulation of negative emotion should occur in response to each positive film.

To index self-reported emotion, participants used a 1–7 Likert scale to rate their negative (fear, sadness, disgust, guilt, distress, anger) and positive (happiness, enjoyment, amusement, affection, relief) emotion immediately following each film. To objectively index emotional facial behavior, participant expressions were coded by five research assistants naïve to study details. Coders rated global indicators of negative and positive emotional expressions to each film [87]. Coders were sufficiently reliable (average ICC = .80, range .74-.90) and ratings were averaged across coders by participant to increase reliability. Reported and coded emotion scores were standardized to z-scores and combined yielding one score for negative emotion during the positive films. Participants also had scores for positive emotion during both films.

### Data analytic strategy

In both studies, the analyses specifically targeted the inhibition of emotional content in order to examine group differences based on genetic variation and interaction. In all analyses we controlled for reported depression because of considerable prior evidence demonstrating a strong link between depression and emotion-inhibitory processing [88] and our interest was in isolating genotypic influences on these processes. In each of Study 1 and Study 2, we performed omnibus ANCOVA tests. In Study 1, we isolated reaction times for emotion words (negative or positive) by controlling reaction times to neutral words as in previous research in the Emotion-word Stroop task [89,90]. In Study 2, we isolated negative emotion responses that lingered during explicitly positive films that followed negative films in a naturalistic emotion task. In this case, by controlling for negative emotions that were generated in response to the preceding negative films, we were able to isolate the degree to which participants could naturalistically inhibit or down-regulate negative emotion. Analyses of these polymorphisms followed literature convention, [91–95] by comparing Met carriers (Val/Met and Met/Met genotypes) with Val/Val homozygotes for BDNF Val^66^Met, and by comparing T carriers (T/G and T/T genotypes) with G/G homozygotes for *TPH2* rs4570625. Consequently, this resulted in 4 possible genotype combinations for combinatorial analyses: 1) Val/Val—G/G; 2) Val/Val—T carrier; 3) Met carrier—G/G; 4) Met carrier—T carrier. Significance was set *a priori* at p<0.05. All data are graphed as mean ± S.E.M.

## Results

All data underlying the findings described in the current manuscript are fully available to the scientific community. Full access to data can obtain by contacting the corresponding author through email.

### Frequency of rs4570625 and BDNF Val^66^Met genotypes

As shown in **Table 1**the frequency of the rs4570625 alleles (T/T = 17 (7.26%); T/G = 81 (34.62%); G/G = 136 (58.12%) did not differ from Hardy-Weinberg equilibrium, χ^2^ = 1.03, p = 0.31. The frequency of BDNF Val^66^Met alleles (Val/Val = 156 (66.67%); Val/Met = 71 (30.34%); Met/Met = 7 (2.99%) also did not differ from Hardy-Weinberg equilibrium, χ^2^ = 0.10, p = 0.75. N’s for each genotype interaction are also shown in **Table 1**for Study 1 and Study 2. Combined analysis of the TPH2 and BDNF polymorphisms do not appear to be in linkage disequilibrium (D′ = -0.21, r^2^ = 0.01) [96].

**Table 1:** 

Study 1						
		**TPH2 rs4570625 Genotype**				
		G/G	G/T	T/T	Row Total	Table Total
**BDNF rs6265 Genotype**	ValVal	48	30	6	84	
	ValMet	22	8	2	32	
	MetMet	2	1	0	3	
Column Total		72	39	8	119	
Table Total						
Study 2						
		**TPH2 rs4570625 Genotype**				
		G/G	G/T	T/T	Row Total	Table Total
**BDNF rs6265 Genotype**	ValVal	35	32	5	72	
	ValMet	28	8	3	39	
	MetMet	1	2	1	4	
Column Total		64	42	9	115	
Table Total						

### Study 1: Emotion-word Stroop task

**Fig 1:**
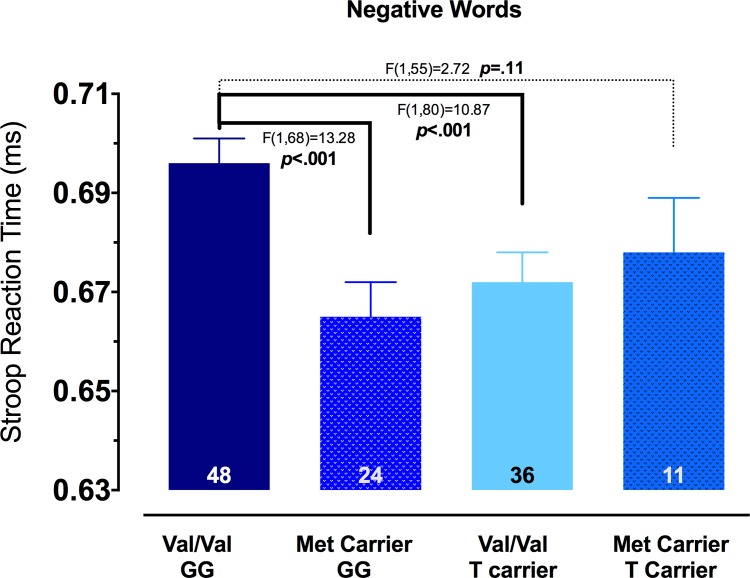
Reaction time to negative words in the Emotion-word Stroop task. Subjects from Study 1 that were Val/Val–G/G had significantly increased reaction times to negative words compared to Val/Val–T Carriers (F(1,80) = 10.87, p<0.001) and Met Carrier–G/Gs (F(1,68) = 13.28, p<0.001). There was also a trend for an increased reaction time of Val/Val–G/Gs compared to Met Carrier–T Carriers (F(1,55) = 2.72, p = 0.11). Met Carrier–G/Gs were not significantly different from Val/Val–T Carriers (F(1,56) = 0.11, p = 0.74) nor from Met Carrier–T Carriers (F(1,31) = 0.70, p = 0.41). Val/Val–T Carriers were also not significantly different from Met Carrier–T Carriers (F(1,43) = 0.22, p = 0.64). All data are presented as mean ± SEM.

We first performed omnibus tests for group differences (BDNF Val/Val vs. BDNF Val/Met or Met/Met; TPH2 G/G vs. TPH2 T/G or T/T; and their combinations) in inhibitory responses to negative or positive emotion words using an ANCOVA, controlling for responses to the neutral words and reported depression. As predicted, we found a significant interaction, *F*(1,113) = 6.02, *p* = 0.016, for negative words. Follow-up tests revealed significant differences by allelic combination. Surprisingly, individuals with the Val/Val–G/G combination showed the weakest inhibition of negative content (i.e., slowest response time for negative words), relative to Val/Val–T carriers, *F*(1,80) = 10.87, *p* < .001, and Met carrier-G/Gs, *F*(1,68) = 13.28, *p* < .001, but were not significantly different from Met Carrier–T’s, *F* (1,55) = 2.72, p = 0.11. (See Fig 1 for all group comparisons). There were no individual main effects for genotype for either the BDNF polymorphism nor the TPH2 polymorphism for either negative or positive words, and no interaction for positive words.

### Study 2: Naturalistic emotion-inhibition

**Fig 2:**
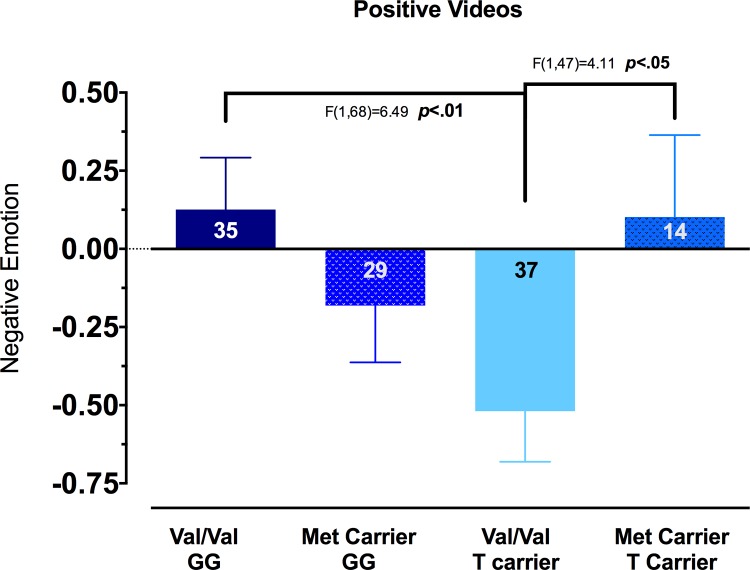
Negative emotions expressed during positive contexts. Reported and coded emotion scores from subjects in Study 2 were standardized to z-scores and combined yielding one score for negative emotion during the positive films. Negative emotion of Val/Val–T Carriers was significantly lower than those exhibited by Val/Val–G/Gs (F(1,68) = 6.49, p<0.01) and by Met Carrier–T Carriers (F(1,47) = 4.11, p<0.05). Negative emotion of Val/Val–T Carriers was not significantly different from Met Carrier–G/Gs (F(1,62) = 1.75, p = 0.19). Val/Val–G/Gs were not significantly different from Met Carrier–G/Gs (F(1,60) = 1.65, p = 0.20) nor from Met Carrier–T Carriers (F(1,45) = 0.02, p = 0.90). Met Carrier–G/Gs were also not significantly different from Met Carrier–T Carriers (F(1,39) = 0.96, p = 0.33. All data are presented as mean ± SEM.

As in Study 1, we first analyzed for group differences (BDNF Val/Val vs. BDNF Val/Met or Met/Met; TPH2 G/G vs. TPH2 T/G or T/T; and their interaction) in inhibitory processing of negative emotion using an omnibus ANCOVA test, controlling for reported depression as well as emotion responses to the previous films. Specifically, we tested to see if there were group differences by genotype and interaction in negative emotion responses during the positive films. Because of the specific parameters of the paradigm, all negative films (eliciting intense negative emotions) were followed by explicitly positive films, placing demands on participants to inhibit negative emotions during the positive films. As such, negative emotions present during positive films would be indicative of poor inhibition of negative emotion. The results of the analysis of negative emotion were highly similar to the results from Study 1. Consistent with our predictions, there was a significant interaction between the two polymorphisms, *F*(1,109) = 5.50, *p* = .02, and no main effects for genotype for either polymorphism. As with Study 1, individuals with the Val/Val–G/G combination showed the weakest inhibition of negative content, demonstrating the highest negative emotion responses during positive films. As before, differences were most clear when compared to Val/Val–T carriers, *F*(1,68) = 6.49, *p* < .01 and also consistent with Study 1, there were no differences with Met Carrier-Ts. (See Fig 2 for all group comparisons).

## Discussion

This is the first report of the potential risk-related consequences of a novel interaction between the BDNF Val^66^Met and TPH2 rs4570625 G-703T polymorphisms. Specifically, in this investigation we were able to detect the influence of a significant interaction between the *TPH2* rs4570625 and *BDNF* Val^66^Met polymorphisms on negative emotion inhibitory processing. We observed consistent evidence across two different emotion-inhibition paradigms, one with high internal validity (Study 1) and one with high ecological validity (Study 2) that the combination of Val/Val and G/G genotypes was associated with impaired inhibition of negative emotional content. This was initially surprising given our original hypothesis that Met Carrier-Ts would exhibit the most pronounced impairments in negative emotion inhibition. Because in neither study did Met carrier-Ts differ from the Val/Val-G/Gs, it remains plausible that the former individuals are also at increased risk. There is growing consensus that inhibitory processes (regulating attention, behavior, and memory) are an essential component of emotional systems that influence nearly all psychological processes [97–103]. The ability to inhibit emotional responses or emotional information is well-recognized as a significant predictor and characteristic of common affective disease, including depression [88], post-traumatic stress disorder [104,105] and anxiety disorders [106]. Moreover, current evidence suggests that poor inhibition of negative emotion can reliably predict the onset of pathology following a stressful life event [87] as well as its persistence over time [75].

The current results indicate that the interaction of two polymorphisms–*BDNF* (Val^66^Met) and *TPH2* (rs4570625; G-703T)–yields two at-risk groups, both characterized by significantly greater difficulty inhibiting negative emotional content. This was most clearly evident when the BDNF–Val/Val was combined with the TPH2 –G/G, but also BDNF–Met was combined with TPH2-T. This pattern is most evident in Study 1, but is effectively replicated in Study 2 (in a more naturalistic task), providing further evidence that these two genotype combinations contribute to high-risk patterns of emotion-inhibitory processing across measurement or methodological contexts.

When studied in isolation, the BDNF Val^66^Met Met allele has been associated with reduced hippocampal volume, poor performance on hippocampal-dependent tasks in humans [40,93,107,108] and thus has been considered a risk allele. However, it is also associated with some cognitive benefits, including improved performance on executive tasks with less hippocampal involvement [109–111], also consistent with our findings here. Collectively, these data suggest that mixed evidence of performance deficits associated with unique alleles may be task-specific and substantial epistatic relationships may exist between BDNF and genes involved in serotonin regulation. This could result in complex effects on emotion inhibitory processing and risk for psychiatric disease–findings that would not be predicted or detected if each gene was studied in isolation.

Although only limited prior data are available, the functional effect of the TPH2 rs4570625 polymorphism may be to alter DNA-protein interactions, ultimately affecting transcription of the *TPH2* gene, as the presence of the T-allele is associated with reduced *TPH2* promoter activity *in vitro* [26,27]. This is hypothesized to result in reduced serotonin levels, although this has yet to be confirmed *in vivo*. In two studies, the rare T/T genotype was associated with poor impulse control and decreased executive control [29,111]. Other studies have found that the T allele is predictive of amygdala and cortical responses to emotional content [28,32–34,112] and has been significantly associated with major depressive disorder [113]. Yet, similar to the BDNF Val^66^Met Met allele, the TPH2 T/T genotype has also been associated with significantly lower measures of trait anxiety [114,115] suggesting a trade-off between negative emotionality and cognitive functions. In the present study, however, only when the G/G genotype of rs4570625 was combined with Val/Val of BDNF Val^66^Met did we find impaired inhibition of negative emotional content, indicating a more intricate interaction between BDNF and TPH2 genes. Taken together, these findings suggest that the poor emotion-inhibitory responses exhibited by Val/Val-G/Gs and Met Carrier-Ts may leave them particularly vulnerable for the most common emotion-related psychiatric disorders.

Recent studies in humans and mutant mouse models provide supporting evidence for biological epistasis between BDNF and genes involved in serotonin regulation [11,12,46,47,51] that may alter neural circuitry and thereby influence risk for psychiatric disease. For instance, the BDNF Val^66^Met Met allele has been found to ameliorate differences in limbic circuitry associated with the 5-HTTLPR S allele [11]. Thus, reductions in BDNF may minimize the increased serotonergic tone hypothesized to be associated with reduced serotonin transporter function. Indeed, researchers are continuing to uncover the lifelong emotional consequences of dysregulated serotonergic signaling (particularly during neurodevelopment) [57–62]. For example, constitutive genetic or transient early life pharmacologic increases in serotonin signaling in rodents have been associated with increased anxiety- and depressive-like behaviors [116,117] as well as social impairments and stereotypy [118,119]. Though the physiological characterization of TPH2 rs4570625 is limited [26,27], such research supports our findings here, indicating that homozygosity for *both* the higher producing BDNF allele (Val) and the higher producing TPH2 allele (G) might contribute to a serotonergic tone that falls outside an optimal range. These findings are also supported by evidence across the two studies that presence of *both* the lower producing BDNF allele (Met) and the lower producing TPH2 allele (T) was linked to impairments in negative emotion inhibition that did not significantly differ from those of the at-risk Val/Val-G/G group. Indeed, it is the genotype groups with a *combination* of one lower producing allele and one higher producing allele that did not exhibit such pronounced negative emotion inhibition deficits. These particular genetic combinations (G/G and Met carrier, or T carrier and Val/Val) may, in combination, help maintain serotonergic tone within an optimal range, possibly during neurodevelopment.

There are some limitations to our study. The first of these being sample sizes that were constrained by the labor-intensive nature of assessing this complex behavioral phenotype. However, the benefit of this approach, which includes more objective and valid indicators of emotion-inhibitory processing, outweigh the limitations of small sample sizes. Our subject pool consisted of an overwhelming majority of Caucasian individuals, preventing evaluation of race- or ethnicity-specific stratification of effects. This shortcoming could be addressed by replication of these methods in regions without a predominant Caucasian presence. Additionally, the Met Carrier-Ts group itself was small in both studies, an inherent limitation due both to the overall sample sizes as well as the much lower frequency of these Met and T alleles. In addition, we focused our study on negative emotion inhibitory processing elicited in-lab. Future studies would benefit from including environmental and life-history data given the importance of gene × environment interactions on risk for psychiatric disease. Though our analyses indicate that the TPH2 and BDNF polymorphisms are not in linkage disequilibrium, there remains the possibility that we are unintentionally measuring the effects of an unidentified gene or polymorphism that is in linkage disequilibrium with one or both of these polymorphisms.

## Conclusion

This investigation makes an important contribution to the understanding of the genetic basis of vulnerability to the most common emotion-related psychiatric diseases. In particular, ours is the first study to suggest an epistatic relationship between BDNF Val^66^Met and TPH2 rs4570625 polymorphisms for impaired regulation of negative emotional content, which has been consistently linked to common affective disorders. These data also emphasize the importance of considering multiple genes and their relationships when investigating their genetic influence on complex behavioral outcomes. Integrating multiple genetic influences on prominent underlying risk factors for emotional psychiatric diseases, such as poor inhibition of negative emotion, rather than associations with broader disease states, will help build more comprehensive models and identify groups at risk for psychiatric disease.
